# The contributions and future direction of Program Science in HIV/STI prevention

**DOI:** 10.1186/s12982-018-0076-8

**Published:** 2018-05-28

**Authors:** Marissa Becker, Sharmistha Mishra, Sevgi Aral, Parinita Bhattacharjee, Rob Lorway, Kalada Green, John Anthony, Shajy Isac, Faran Emmanuel, Helgar Musyoki, Lisa Lazarus, Laura H. Thompson, Eve Cheuk, James F. Blanchard

**Affiliations:** 1 Centre for Global Public Health, College of Medicine, Faculty of Health Sciences, University of Manitoba, Winnipeg, Canada; 2 Li Ka Shing Knowledge Institute, St. Michael’s Hospital, Toronto, Canada; 3 Division of Infectious Diseases, Department of Medicine, Faculty of Medicine, University of Toronto, Toronto, Canada; 4 Division of STD Prevention, The National Center for HIV/AIDS, Viral Hepatitis, STD and TB Prevention, Centers for Disease Control and Prevention, Atlanta, USA; 5 Karnataka Health Promotion Trust, Bangalore, India; 6 National AIDS and STI Control Program, Ministry of Health, Nairobi, Kenya

**Keywords:** Program Science, HIV prevention, STI prevention, Public health programs

## Abstract

**Background:**

Program Science is an iterative, multi-phase research and program framework where programs drive the scientific inquiry, and both program and science are aligned towards a collective goal of improving population health.

**Discussion:**

To achieve this, Program Science involves the systematic application of theoretical and empirical knowledge to optimize the scale, quality and impact of public health programs. Program Science tools and approaches developed for strategic planning, program implementation, and program management and evaluation have been incorporated into HIV and sexually transmitted infection prevention programs in Kenya, Nigeria, India, and the United States.

**Conclusion:**

In this paper, we highlight key scientific contributions that emerged from the growing application of Program Science in the field of HIV and STI prevention, and conclude by proposing future directions for Program Science.

## The beginning of Program Science

**Fig. 1:**
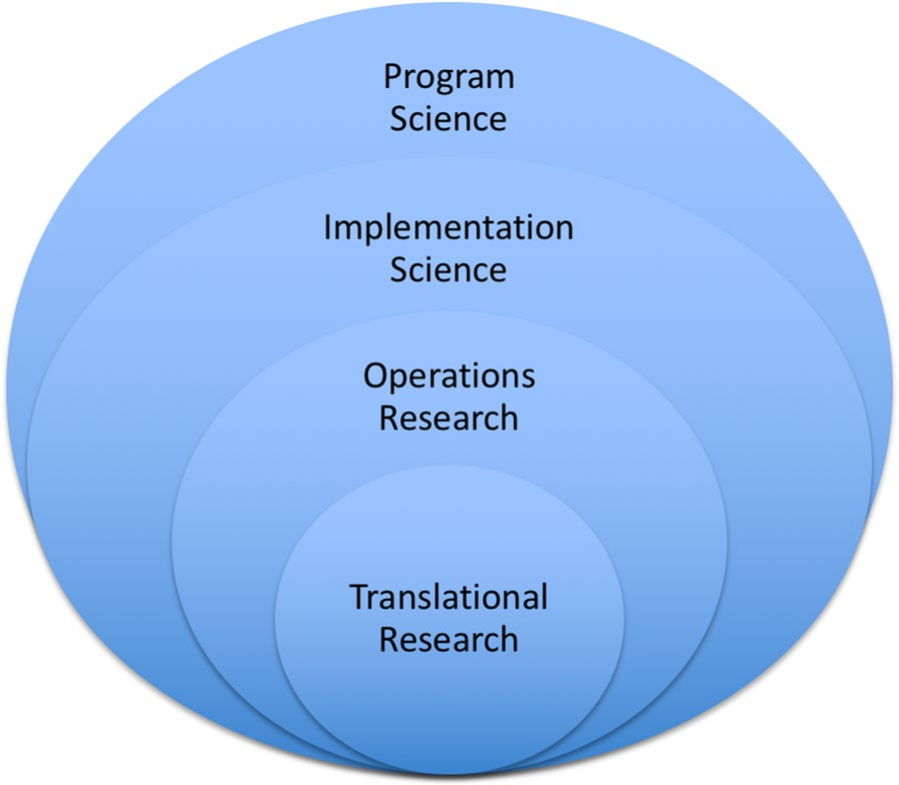
Program Science and its relationship with other research frameworks

The field of Program Science was introduced to the scientific community and applied as a novel framework for generating new knowledge for—and from—HIV and sexually transmitted infection (STI) prevention programs [1, 2]. Program Science is defined as the systematic application of theoretical and empirical knowledge to optimize the scale, quality and impact of public health programs [1]. The Program Science initiative draws on and encompasses many key elements of other research frameworks, including Implementation Science [3, 4], Operations Research [5] and Translational Research [6] to answer critical programmatic questions (as illustrated in Fig. 1). While there is overlap with all of these frameworks, one of the distinguishing features with Program Science is its’ bidirectional approach. At the core of Program Science is the principle of getting research *out* of programs and *into* practice [7], whereas the other frameworks focus on understanding how best to implement an intervention.

Program Science was conceptualized in response to challenges encountered at the interface of research and programs in HIV/STI prevention, where there remained a disconnect in the perspectives and priorities of scientists, program implementers and policy makers [1, 8, 9]. Program Science was conceived as an iterative, multi-phase research and program framework, within which scientists, program implementers, and policy makers work together [1, 2] so that practice informs research and research informs practice and policy [7]. This strategy fosters an adaptive response which enables programs to continuously and systematically examine its’ program processes, outputs and outcomes and then use this new knowledge as described below.

## Three spheres of Program Science

**Fig. 2:**
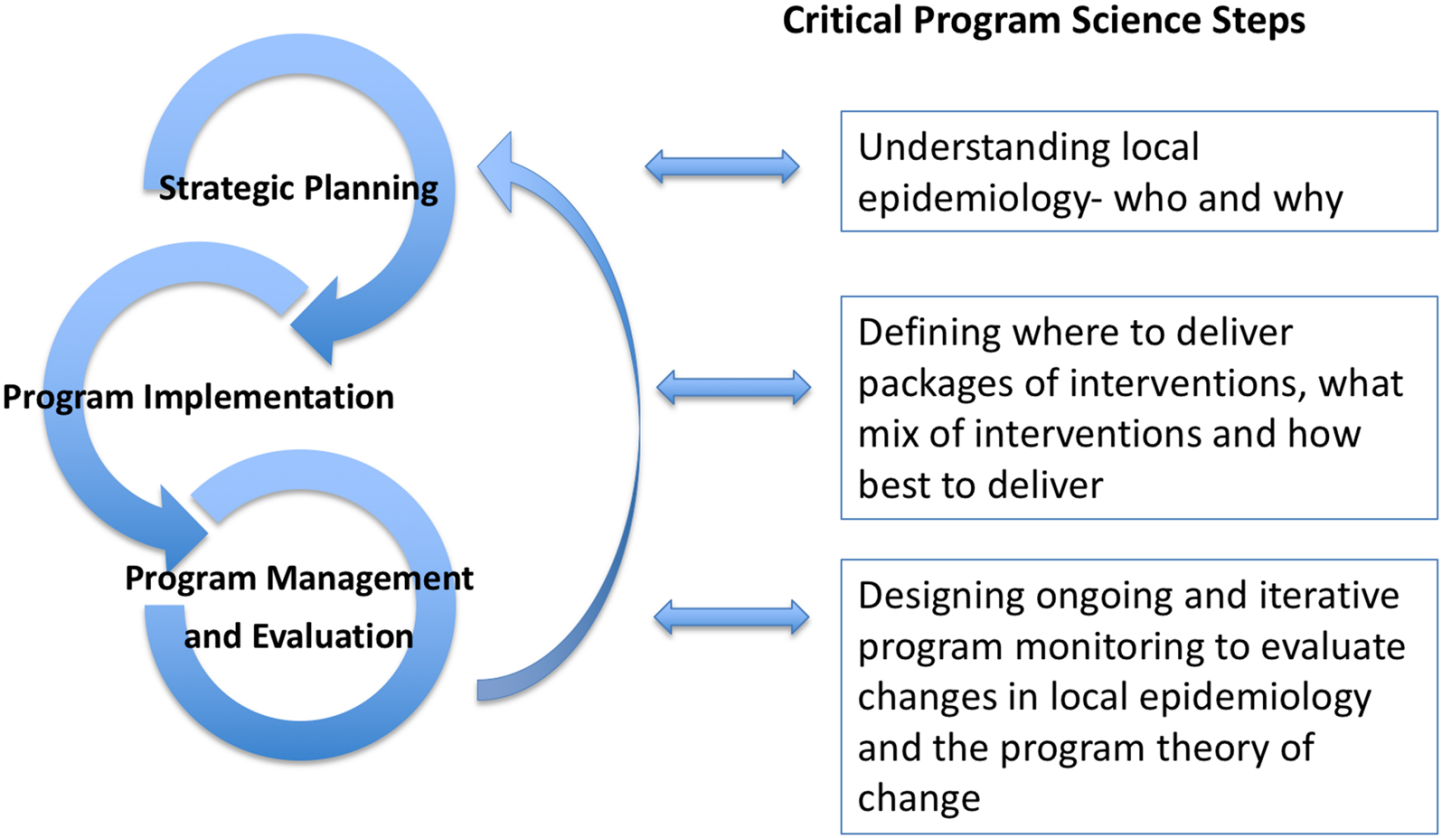
The three key spheres of a Program Science cycle and illustrations of critical steps within each sphere

The three spheres of a program cycle include: (1) strategic planning; (2) program implementation; and (3) program management and evaluation (see Fig. 2) and these form the basis for the application of Program Science. By encompassing these three spheres of a program cycle, Program Science, as both a program and research framework, is able to ensure that scientific enquiry is driven by these spheres, and the subsequent application of the knowledge generated from scientific enquiry, systematically addresses all three spheres.

The strategic planning sphere of a program cycle centers on making informed decisions about program priorities and resource allocation. For example, heterogeneity in risk—through place or geographic location and social determinants—underpin HIV and STI epidemics [10–13]. Thus, epidemic control requires a program aligned with local epidemic context in order to address this heterogeneity [14–16]. The implementation phase of a program cycle centers on making informed decisions about ‘where’, ‘what’, ‘how’, and ‘for whom’ to deliver interventions. Critical decisions for program implementation include the locations for implementation and the populations that will be focused on by the program, the specific combination of interventions to be implemented, as well as how best to deliver these services. Finally, program evaluation requires the generation of robust evidence as part of program management. It is an ongoing and iterative process that allows for the re-development and re-design of programs to respond to program indicators and outcomes and to evolving epidemics, structures and drivers of an epidemic. For example, as a public health program progresses, the knowledge on heterogeneity is then used to fine-tune decisions on the ‘where’, ‘what’, ‘how’, and ‘for whom’ and program monitoring focuses on whether gaps, or inequities, in a program are improving. Program Science supports the generation of knowledge across these spheres in order to inform HIV/STI programs with some examples discussed below.

## Program Science in practice

Programs at the national and sub-national level [1, 2, 17, 18], HIV prevention researchers [19, 20], policy makers [21, 22], and community-based organizations [23, 24] have implemented a Program Science approach to tackle issues of public health importance [19, 20, 25] and this approach has generated important scientific contributions, as shown in Table 1 and discussed here.

**Table 1:** Key scientific contributions of Program Science and future directions

Program Science spheres	Scientific contributions	Future directions
Strategic planning	1. Geographical mapping 2. Hotspots-spatial distribution of epidemics 3. Transmission dynamics	1. Rapid ethnographic assessments and enhanced geographical mapping 2. Micro-level (within city) appraisals of risk clusters 3. Program design by epidemic phase
Program implementation	1. Intervention mix 2. Community engagement and mobilization	1. Delivery platforms for agentic, individual and structural interventions 2. Context specific adaptation
Program management and evaluation	1. Tools for field level monitoring	1. Complex systems evaluation 2. Real time evaluation for responsive adaptation 3. Optimized indicators aligned to program stage

### Strategic planning

For HIV/STI program design, the necessary evidence involves an incisive appraisal of the social and epidemiological drivers and mediators of local epidemics. This includes understanding the places and drivers that might disproportionately place key populations (KPs) at higher risk of HIV/STI acquisition as well as characterizing population-level chains of transmission.

Innovations in *Programmatic Mapping* involves a systematic approach to generating key information about the size and distribution of KPs within a defined geographic area [26]. Other methods, including multiplier methods or capture-recapture techniques, provide overall size estimates but do not provide the granular information required for detailed program planning and implementation. For example, geographic mapping provide city-wide KP size estimates and also provide data on micro-level hotspot (places where KPs congregate to solicit sex/drug using partners) level KP size estimates, as well as generate information on the physical locations where KPs congregate and the characteristics of these locations, such as the typologies of sex work. The detailed population size data allow programs to set coverage goals and the location data enable programs to plan for outreach and concentrate resources in areas of greatest need. Programmatic mapping has been used by many countries in Asia and Africa [27–30] and there is growing global recognition of the importance of mapping data [31]. David Wilson, the World Bank’s Global AIDS Program Director, recently wrote that “programmatic mapping are the foundation for high quality HIV programs” [32].

While programmatic mapping provides data on micro-level geographic concentration of risk, there has also been work to understand the macro-level *spatial distribution* of the epidemic at the province/state/district level as highlighted in work led by Tanser et al. and Abu-Raddad et al. [13, 16]. Tanser demonstrates that in regions where the HIV epidemic was traditionally felt to be a generalized epidemic, that in fact, there were important zones of high HIV transmission signifying the presence of concentrated sub-epidemics. Prioritizing finite resources by place (e.g. province or state) may be more efficient than universal distribution of resources across a country [33] to reduce HIV infections. Similarly, re-allocation of resources to better align service delivery with disease burden and disparities requires detailed mapping of health-states and services, including how individuals navigate health systems [34, 35].

Additional innovations have included approaches for characterizing HIV epidemics by understanding the causal pathway of *HIV transmission* at a population-level rather than focusing on HIV acquisition at an individual level. For example, condomless sex acts in the context of sex work may lead to a small number of HIV infections in the short-term, but contribute to a large number of HIV infections over time through onward transmission [36–38]. Disentangling the causal pathways may require a more in depth understanding of the local context of sex partnerships, which in turn, leads to a better understanding of the sources of heterogeneity in risk of HIV transmission, and of acquisition. For example, the importance of transactional sex (sex in exchange for money/goods/resources wherein exchange was not explicitly negotiated prior to sex) leading to high proportion of HIV acquisition was recognized when a revised Modes of Transmission Model was parameterized to the local Nigerian context [36].

The uptake of some of these innovations into policy for resource allocation can be seen with an example from the Centers for Disease Control and Prevention (CDC). In 2013, the National STD Prevention Program in the United States was revised to incorporate a strategic planning component to its state funding allocation and provides a useful example of the application of Program Science in a northern hemisphere country context. The Division of STD Prevention at the Centers for Disease Control and Prevention in Atlanta is responsible for all of STI prevention in the United States. The funding requirements use a Program Science framework for resource allocation [22], using STI disease burden by subgroup, and subgroup population size, and thereby requiring programs/states to generate local knowledge about STI epidemiology through methods like programmatic mapping.

### Program implementation

The Avahan India AIDS Initiative of the Bill and Melinda Gates Foundation was a large scale focused HIV and STI Prevention Program in South India for KPs. Avahan used programmatic mapping for strategic planning and specifically to determine *where*, *when*, and *for whom* interventions should be prioritized. Avahan is also a very nice example of using Program Science to determine *what intervention mix* is required and *how* to deliver these interventions in their programs [23, 39, 40].

Avahan clearly demonstrated the need to combine behavioural, biomedical and structural interventions to achieve the maximum impact in reducing HIV and STI rates. Biological and behavioural surveys conducted among female sex workers (FSWs) revealed a decline in HIV, syphilis, chlamydia and gonorrhea prevalence in most sex work sub-groups and most locations as a result of combination prevention interventions which included STI prevention and treatment [41–44]. As the program matured, the “*what*” and the “*how*” also evolved. The program began to incorporate structural interventions aimed at reducing violence and improving community mobilization [23]. The inclusion of these interventions was driven by needs voiced by community members (members of KPs) as well as a program aim to further reduce HIV and STI rates. The process of designing and implementing these structural interventions centered on comprehensively engaging with policy makers, police, lawyers, media and sex work communities. With the incorporation of these interventions into the existing multi-pronged prevention programs, reductions in reported violence and improved individual and collective mobilization and empowerment were also seen [45, 46]. These changes also resulted in increases in the number of FSWs accessing government social programs and in some areas, improvements in condom use and service utilization [47]. The use of a Program Science framework allowed for a dynamic response; as the needs of the community changed, the program also evolved, using evidence to reshape and redesign the program and its’ implementation.

An important dimension of Avahan’s effectiveness is the integration of community knowledge in informing its intervention mix. Ashodaya Samithi [44, 48, 49], the first intervention site supported by Avahan, has developed community-centric processes and responses that allow communities to prioritize their issues, set the agenda for the way forward, and ensure community ownership of the intervention. This is achieved at multiple levels, initially through community engagement and involvement, and later through ownership of the intervention and capacity building that ensures sustainability of the intervention [23]. These levels of community involvement have been found to result in communities re-interpreting and translating intervention messaging at the local level to develop contextualized responses to public health challenges [24].

### Program management and evaluation

**Fig. 3:**
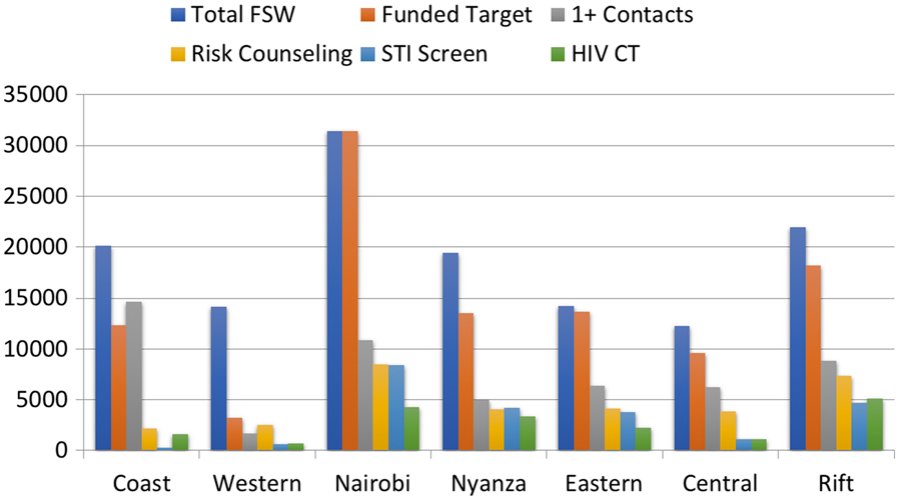
Program monitoring data for HIV/STI Prevention among FSWs in Kenya (April-June 2013)

Improving program efficiency requires an approach to identify and define existing opportunity gaps. The Program Science Initiative in Kenya, through a Technical Support Unit (TSU) to the National AIDS and STI Control Programme (NASCOP), developed innovative field level tools to capture data on HIV/STI prevention program indicators. HIV prevention programs in Kenya follow a combination prevention approach with a focus on biomedical, behavioural and structural interventions. The tools developed and used by these programs were developed to collect data on all aspects of the program covering all three of these intervention focus areas. Kenya, as many other countries do, has several funders of KP programs. As such, implementers were using many different reporting formats used by the many different funders. The TSU, with support from NASCOP, worked with all funders and implementing partners through the National Key Population Technical Working Group to develop standard data collection tools. A basic 15 indicator reporting tool was developed and all implementing partners were mandated to report to NASCOP on a quarterly basis on all 15 indicators [50, 51]. This standard tool was useful to both simplify and harmonize data collection and reporting. The reports are compiled at the national level by TSU and NASCOP and county wise analysis is shared with the implementing partners, county governments and funders on a quarterly basis to: (1) examine data quality; (2) evaluate trends such as changes in HIV testing uptake over time and (3) assess program achievements as compared to national targets. Figure 3 illustrates the layers of data collected and highlights the differences in coverage across the counties in Kenya.

## Future directions for Program Science

Important next steps within Strategic Planning include enhanced geographic mapping along with micro level appraisals. For example, a particular challenge noted by program staff in several countries was the provision of services to young and new FSWs with high rates of HIV acquisition prior to program engagement [52, 53]. Targeted preventive interventions generally reach women only after they self-identified as sex workers [52]. To understand the distribution and population size of young FSW, enhanced geographic mapping which involved micro level (within city) appraisals in Kenya and Ukraine to map locations where young women seek sexual partners, including paid, transactional and casual sex partners [54]. Knowing who, where and how much early HIV risk exists will help refine the design and delivery of programs for FSWs and other vulnerable young women.

Additional future directions for strategic planning involve an adaptive design by the phase of the epidemic (growing, stable, declining) and in the context of baseline and co-existing interventions [55, 56].

Next steps for implementation include resolving tensions between agentic, individual and structural interventions with a focus on optimizing synergies across delivery platforms [57]. Considerable scope remains to advance Program management drawing upon evaluation frameworks and focusing on complex adaptive systems. By treating public health programs as complex systems, opportunities exist for identifying emergent properties and learning through the life course of a program in real time.

## Future directions: expanding the tools

**Fig. 4:**
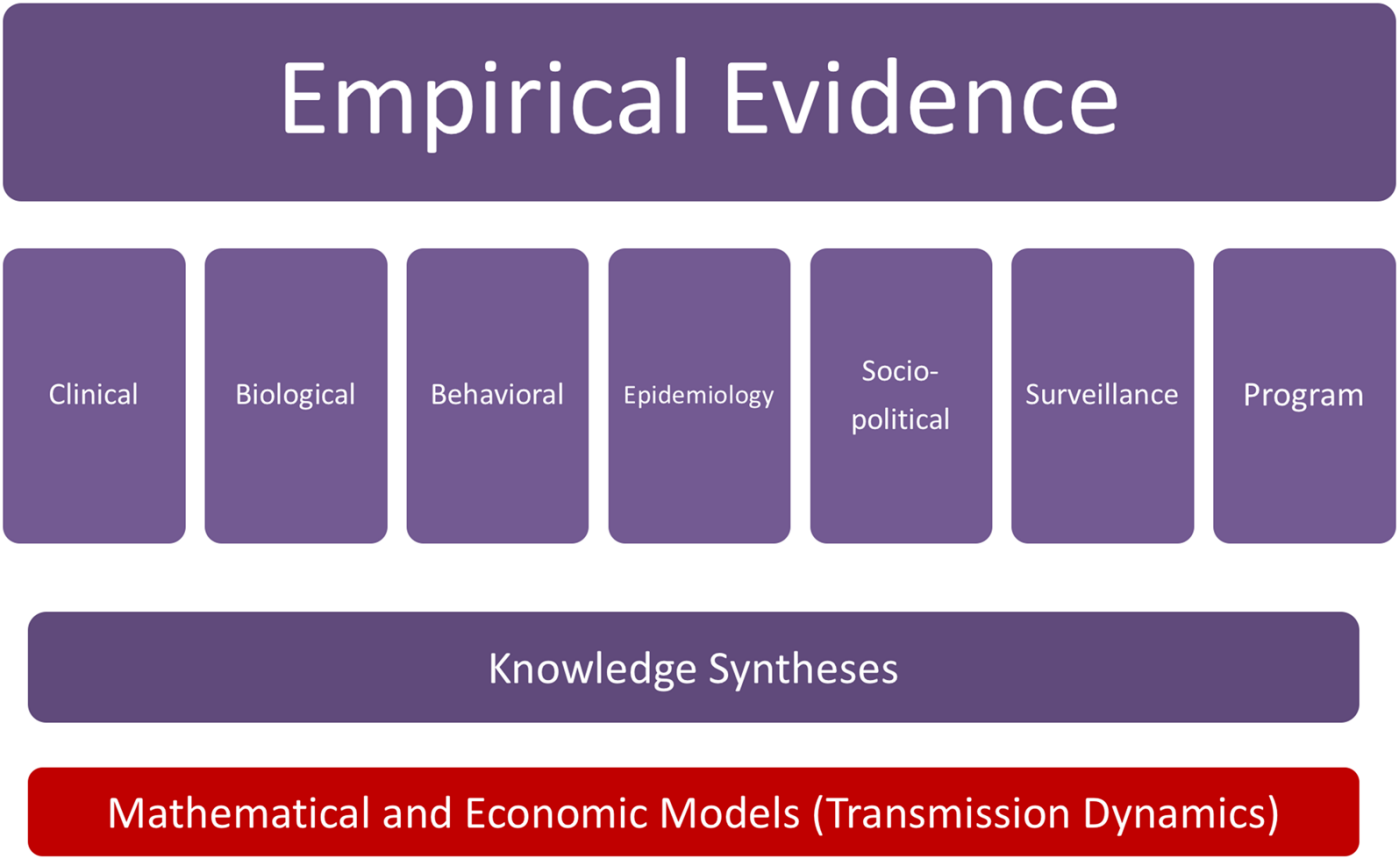
Layers of evidence used within Program Science. Empirical evidence is generated in many forms, including program data. Hypotheses are tested using several methods. A key component of Program Science involves syntheses of knowledge across multiple levels and scope, including realist reviews, and the integration of these data and syntheses with mathematical models to project public health impacts on health and costs. For infectious diseases such as HIV and STIs, public health impacts are estimated using transmission dynamics models

The scientific arms of Program Science comprise a range of methods and disciplines—and most importantly—a multidisciplinary scientific approach. Empiric evidence covers multiple ‘layers’, from the molecular to environmental (Fig. 4), while conceptual frameworks that underpin the science are grounded in socio-behavioural [58, 59], complexity, and mathematical theory [60, 61].

Future expansions of the Program Science toolbox include the development of new mathematical models with novel applications; effective data visualization tools for program monitoring to reflect complex interactions; analytic frameworks to integrate multiple layers of biological (host and pathogen) and behavioural data to disentangle causal pathways to population-level transmission; resource allocation tools that incorporate explicit trade-offs within programs, health-systems, and communities.

Finally, expansion of Program Science includes the development of a Community-Based Program Science framework which draws on scaling up the principles of participatory engagement.

## Conclusion

Program Science is an emerging field in public and population health. Through the country examples, this paper highlights some of the important scientific contributions that have developed over the past 5 years. Program Science as a framework is unique among other research strategies because it systematically combines the program cycle with the research strategy by embedding research within programs and having programs set and drive the research agenda. This approach requires partnership between policy makers, program leaders, service providers, researchers and communities. This combined effort results in a focus on ensuring maximum population level benefit of a program through detailed understanding of the local needs and context. This strategy has the potential to close the gap between evidence, action and policy and may be applicable to many important public health areas globally.

## Abbreviations

CDC: Centers for Disease Control and Prevention
FSW: female sex worker
HIV: human immunodeficiency virus
KP: key population
NASCOP: National AIDS and STI Control Programme
STD: sexually transmitted disease
STI: sexually transmitted infection
TSU: technical support unit
